# Community Pharmacistsꞌ Role in Controlling Bacterial Antibiotic Resistance in Aleppo, Syria

**Published:** 2017

**Authors:** Ossama Mansour, Rawaa Al-Kayali

**Affiliations:** a *Faculty of Pharmacy, Al Andalus University for Medical Sciences, Tartuas, Syria. *; b *Department of Biochemistry and Microbiology, Faculty of Pharmacy, Aleppo University, Aleppo, Syria. *; c *Public Health Department, Aleppo University Center for Strategic Studies, Aleppo, Syria.*; 1 O. S. and R. A. contributed equally to this work.

**Keywords:** Antibiotics resistance, Community pharmacy, Knowledge, Attitude, Aleppo, Syria

## Abstract

Antibiotic resistance is a major public health concern. This study was conducted to evaluate the knowledge, attitudes of community pharmacists regarding antibiotic use and potential drug resistance besides assessing their behaviors about dispensing antibiotic without prescription and correlation of the outcomes with demographic variables. A cross-sectional survey was conducted on a random sample of 250 pharmacies in Aleppo, using validated self-administered questionnaire. The total scores of the pharmacistsʹ knowledge and attitudes were correlated with participant demographics using Chi-squared test. One-hundered-seventy-three pharmacies out of 250 agreed to participate in the study. The overall prevalence of dispensing antibiotic without prescription was 85.5%. Only 30.8% of participants exhibited good attitude and 37% had adequate knowledge about antibiotic resistance. Good attitude was strongly correlated with age (*P *= 0.023), years of experience (*P *= 0.007), socioeconomic location of the pharmacy (*P *= 0.009) and number of clinics near pharmacy (*P *= 0.008). The results of this study confirmed that dispensing antibiotic without prescription is a common practice in Aleppo pharmacies despite being unlawful. The half of community pharmacists has a poor attitude and inadequate knowledge with regard to antibiotic resistance, reflecting the need for awareness-raising campaigns directed to community pharmacists to equip them for their main role in the community.

## Introduction

The role of the pharmacist has been changing over the past two decades. He is not just a supplier of drugs and a distributor of medicinal products, but also a team member involved in the provision of healthcare (1, 2). Community pharmacists with the advantage of their accessibility to patients in the community could play an intrinsic role to reduce the rise of antibiotic resistance by advising patients not to use antibiotics for such self-limiting infections and help play their part in a concerted effort to reduce the rise of antibiotic resistance (3).

The major cause of antimicrobial resistance is the inappropriate use of antibiotics, which was found to be directly related to the tendency towards self-medication and the unnecessary use of antibiotics for viral disease (4).

In this connection, WHO report (2014) emphasized on examination and, where necessary, the improvement of the role of the pharmacist as the main supplier and regulator of antibiotics (5). Most initiatives regarding antibiotic misuse are directed toward optimizing physiciansʹ prescriptions, while other potential sources of antibiotic misuse are neglected (6). However, the effectiveness of antibiotics in the community and the risk of resistance may also be influenced by how antibiotics are used by the patient (7). Antibiotics, while often classified as prescription-only medicines, can be purchased without prescription from various drug outlets and community pharmacies in a range of countries around the world (8). Despite being illegal, over-the-counter sales of antibiotics occur frequently in Syria. The supply of an antibiotic from a pharmacy without a prescription usually involves a consultation with a pharmacist (9, 10). Therefore, modifying public attitudes and improving the knowledge of the people regarding antibiotic use will be a responsibility of community pharmacist, the source of these drugs (11, 12). Pharmacies in low- and middle-income countries are often the first point of contact for patients seeking health care as they are usually more accessible and less socially distant than other providers, including medical doctors (13, 14). However, staff at these shops do not always recommend appropriate or adequate medicines or treatment regimens, causing concerns about public health issues such as antibiotic resistance (15).

Pharmacistsʹ knowledge and attitude on antibiotics related issues can greatly influence the way this drug is used in. So, this study aimed at explore pharmacists’ knowledge, attitudes, and dispensing habits with respect to antibiotics and microbial resistance, in view of the potential link between these and the practice of dispensing of antibiotics without a medical prescription.

## Experimental

*Study design*

This was a cross-sectional study conducted among community pharmacies from Aleppo, Syria. Pharmacies included in the study were identified from database of pharmacists syndicate. A pretested, revalidated, self-administered questionnaire was used to collect data.

*Study tool*

A random selection was performed of 250 pharmacy from a total of 1700 located in Aleppo city based on the geographical location to be representative of all pharmacists.

A pharmacy student visited each selected pharmacy and invited community pharmacists to participate in the study after explaining the aims of the study, participants were told that all information provided was completely confidential and the results would be presented anonymously. During first visit, a self-administered questionnaire which was attached with the consent form was given to each participant. In the second visit, the filled questionnaires were collected from pharmacists who accepted to participate in this study. The questionnaire designed to assess the current knowledge, attitudes and towards bacterial resistance to antibiotic. The questionnaire comprised 21 questions. The first part consisted of two questions; one closed-ended and one open-ended, designed to investigate community pharmacistsʹ behavior toward dispensing antibiotics without prescription for tonsillitis (as example for infectious disease) and possible reasons behind it. The second part consisted of ten questions; five open-ended and five close-ended, which used a three-point scale (frequently, sometimes, never). This part is about exploring the pharmacistʹs attitude toward providing guidance during dispensing antibiotics. In the third part, pharmacists’ knowledge about antibiotic resistance was measured with twelve three-point scale (agree, unsure, disagree) questions. Finally, the fourth part is about pharmacist demographic characters. The questionnaire was designed in Arabic then further retranslated into English. The actual survey was conducted during a 2-month period from June to July 2012.

*Statistical analysis*

After completion of data collection, it was reviewed, organized, tabulated and entered into Statistical Package for Social Sciences (SPSS Inc., Chicago, IL) for windows version 18. Descriptive statistics were used to analyze the data (frequency and percentages; mean, standard deviation). The knowledge scores were computed by scoring 1 for each correct answer and 0 for an incorrect answer with ʹunsureʹ added into incorrect. The attitude scores were computed by scoring 1 for each good behavior and 0 for poor behavior with ʹsometimesʹ added into poor behavior. These scores were then summed up and divided by the total number of test items. A score greater than 70% of the possible maximum score was considered as adequate and good and less than 50% as inadequate or poor for knowledge and behavior respectively, whereas a score between 50 and 70% was considered as moderate. 

Statistical associations of demographic variables (gender, age, years of experience, place of graduation, pharmacy socioeconomic area and number of clinics near pharmacy) and total score of knowledge and attitude were examined by pearson’s chi-squared test. Association was established at *P *= 0.05.

## Results

Of the 250 pharmacy selected, 27 (13.8%) were working without a pharmacist and 9 (4%) were not located after two attempts. Of the 214 pharmacists met, 41 (17.6%) refused to participate, giving a response rate of 80.8%. The majority (70.5%) of the community pharmacists were males predominantly graduated from Syrian universities. The mean age of the participated pharmacists was 39.8 ± 10.2 (range 25- 65) years old and the average years of pharmacy working experience were 14 ± 7.6 years (range 2-30 years). The other demographic data of participants were shown in Table 1.

*Dispensing antibiotics without medical prescription*

**Figure 1:**
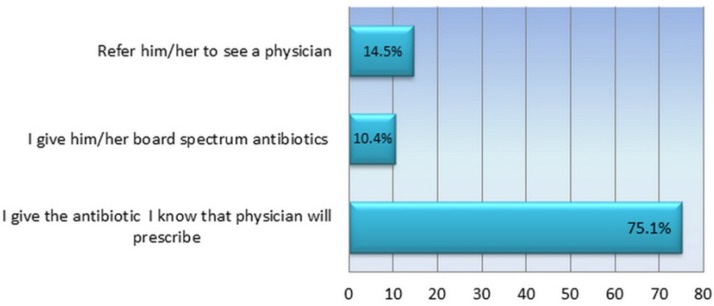
How do you react, if a patient comes to you in the pharmacy asking for antibiotic to treat his tonsillitis?

Participants were asked to answer the questions about speculating case of tonsillitis to determine the percentage of pharmacists who sell antibiotics without a medical prescription. The majority of pharmacists admitted that they dispense antibiotics for tonsillitis treatment without physician prescription (Figure 1), 10.7% of them give the patient a board spectrum antibiotic to eliminate infection, whereas 74.9% said that they choose the antibiotic usually prescribed by physician. Three possible reasons were mentioned by pharmacists to justify their behavior; 12.3% of them explained their attitude that the patient is known to have no money for private physician, 23.9% claimed that he had sufficient information about infection and antibiotics, whereas 63.8% of them explained their behavior as «if I did not give the customer what he wants he would get it from another pharmacy» .The comparison of dispensing behavior among participants showed no association between any demographic characters and their behaviour. Although from pharmacists who graduated from America and western Europe reported that they refer the patients to see a physician compared with 33.3% of them giving the antibiotic without prescription, this difference did not reach statistical significance (*P *= 0.008) Table 1.

*Pharmacistsʹ attitude*

**Figure 2:**
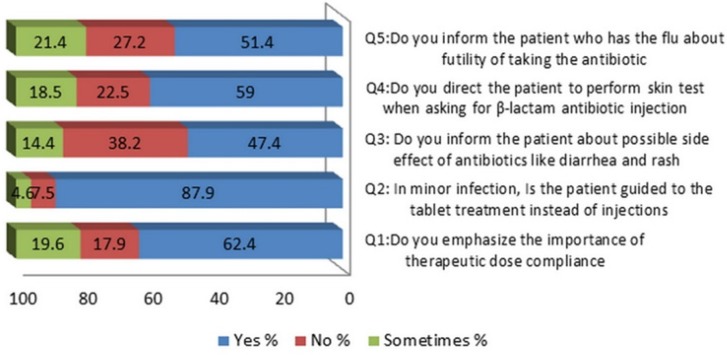
Community pharmacist attitude during dispensing antibiotics

To evaluate pharmacist behavior and role in directing the customer to optimal use of antibiotics, five questions were designed; the pharmacistsʹ answers were summarized in Figure 2. Only (62.4%) of the participated pharmacists emphasize on the importance of therapeutic dose compliance, less than half of them inform patients about useless of taking antibiotics when catching a flu, the others (who has answered

with no or sometimes) explained their behavior as they try to prevent bacterial secondary infection. About side effects of antibiotics, (47.4%) of respondent inform the patients about the possible incidence of diarrhea and rash after taking some antibiotics and 58.8% direct the patient to perform hypersensitivity skin test when asking for β-lactam antibiotic injection. Notably, the majority of pharmacists (87.9%) guided the patients to take antibiotic oral capsule treatment instead of injections in the case of minor infections. The pharmacistsʹ attitudes were classified as good, moderate and poor depending on the scores they gained. The results showed that (30.8%) of pharmacists exhibit a good attitude toward guiding customers, whereas (49.1%) and (20.1%) showed moderate and poor attitudes respectively. Four variables (age, years of experience, socioeconomic location of the pharmacy, number of clinics near pharmacy) showed a significant association with the participants’ attitudes according to the chi-squared test Table 1.

*Pharmacistsʹ Knowledge*

**Figure 3:**
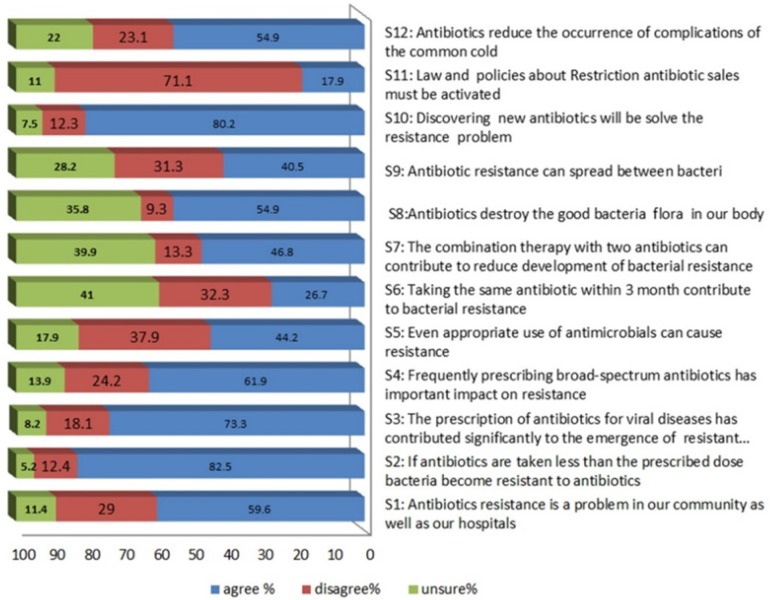
Community pharmacist's knowledge about antibiotic and resistance

Pharmacists’ knowledge about antibiotic resistance was evaluated by using twelve questions as shown in Figure 3. The majority of pharmacists 89.6% feel that antibiotic resistance is a problem in our community, they believed that taking a sub optimum dose of antibiotics (92%), frequently prescribing broad-spectrum antibiotics (81%), and to a lesser extent the prescription of antibiotics for viral diseases (73%) were very important contributors to bacterial resistance. The factors perceived to be minimally important included the use of antimicrobials appropriately (34%), taking the same antibiotic within 3 months (27%).

On the other hand, only 47% think that combination therapy with two antibiotics can reduce resistance development and 90% consider that discovering new antibiotics will solve the resistance problem. Unexpectedly, 71% of participating pharmacists were disagreeing about activation of law that prevents antibiotic sales without prescription. The pharmacistsʹ knowledge was classified as adequate, moderate and inadequate depending on the score they gained. The results showed that (37%) of pharmacists have adequate knowledge, whereas (57.8%) and (5.2%) showed moderate and inadequate knowledge respectively. No association has been found between demographic characters and pharmacists knowledge scores regarding antibiotic resistance according to the chi-squared test Table 1.

## Discussion

Irrational use of antibiotics and weak regulatory enforcement of drug sales are serious issues in developing countries that contribute significantly to bacterial resistance (16). Knowledge and attitude on antibiotics related issues can greatly influence the way this drug is used. Therefore, evaluation of pharmacists dispensing behavior of such drug may provide valuable information which could help towards developing interventions targeting to improve the use of antibiotics (17). 

**Table 1:** Association of participant demographic characteristics with their behavior, attitude and knowledge level.

**χ** ^2 ^ **(** ***P*** **)**	**Pharmacists knowledge level** **(%)**			**χ** ^2 ^ **(** ***P*** **)**	**Pharmacist attitude** **(%)**			**χ** ^2 ^ **(** ***P*** **)**	Dispensing antibiotics** behavior (%)**			**Total N.** **(%)**	**Variable**
	**Adequate** **(%)**	**Moderate** **(%)**	**Inadequate** **(%)**		**Good** **(%)**	**Moderate** **(%)**	**Poor** **(%)**		C*** **(%)**	B** **(%)**	A* **(%)**		
													Gender
0.82	35.2	60.7	7.8	0.86	23.5	45.1	31.4	0.25	3.9	88.2	7.8	51 (29.4)	Female
	34.4	64.1	4.1		19.7	50.8	29.5		18	69.7	12.3	122 (70.5)	Male
													Age group
	28.4	68.7	3		22.4	52.2	25.4		14.9	73.1	11.9	67 (38.7)	25-34
0.79	46.7	48	5.3	0.023	32	46.7	21.3	0.79	9.3	84	6.7	75 (43.4)	35-50
	32.3	58.1	9.7		41.9	48.4	9.7		22.6	61.3	16.1	31 (17.9)	> 50
													Country of graduation
	34.5	60.5	5.3		20.4	46.9	32.7		16.8	72.6	10.6	113 (65.3)	Syria
0.62	35.5	61.3	3.2	0.40	25.8	41.9	32.2	0.08	9.7	83.9	6.5	31 (17.9)	Other Arab countries
	50	42.3	7.7		19.2	61.5	19.2		3.8	84.6	11.5	26 (15)	Russia and eastern Europe
	33.3	66.7	0		0	100	0		66.7	33.3	0	3 (1.7)	America and western Europe
													Years of experience
	34.4	64.1	1.6		21.9	51.6	26.2		10.9	78.1	10.9	64 (37)	Less 11
0.37	40.5	52.7	6.8	0.007	28.4	51.4	20.3	0.56	13.5	77	9.5	74 (42.8)	11-20
	34.3	57.1	8.6		48.6	40	11.4		20	65.7	14.3	35 (20.2)	More than 20
													Socioeconomic location of the pharmacy
	36.9	60	3.1		36.9	50.8	12.3		18.5	72.3	9.2	65 (37.6)	Low
0.94	36.5	55.3	8.2	0.006	30.6	44.7	24.7	0.39	12.9	71.8	13	85 (49.1)	Medium
	39.1	60.9	0		8.7	60.9	30.4		4.3	95.7	0	23 (13.3)	High
													Number of clinics near pharmacy
	18.9	78.4	2.7		43.2	40.5	16.2		5.4	83.8	10.8	37 (21.4)	No clinic
0.36	46.8	48.4	4.8	0.08	32.3	43.5	24.2	0.83	21	72.6	6.5	62 (35.8)	1-3 clinic
	37.8	55.4	6.8		21.6	58.1	20.3		12.2	73	14.9	74 (42.8)	More than 3 clinics

This is the first qualitative study to be conducted in Aleppo, Syria with the aim of exploring pharmacists’ knowledge of and perceptions about antibiotic use and microbial resistance. Aleppo is the largest city in northern Syria. It has about 2.2 million inhabitants. The average number of persons per physician is 790 and 1451 per pharmacist according to Central Bureau of Statistics 2011 (18). 

The present findings would be the first step in providing a baseline quantitative data of antibiotics dispensing pattern, knowledge, and attitudes regarding antibiotic resistance among Aleppo pharmacists.

The current study revealed a high prevalence (85.5%) and easy availability of these agents over the counter despite being illegal as ministry of health passed a law (Number 2/T, dated 12/1/1988) determining which drugs could be sold to individuals without medical prescription and that the antibiotics were clearly excluded (19). This finding is lower than results reported from Indonesia 91% (20), Saudi 97.9% (21), and Syrian previous study (10). Whereas, in Lebanese study, the prevalence rate of antibiotics sales over the counter was 32.1% (22). The high observed rate of antibiotic sales without a prescription in Aleppo could be attributed to lack of enforcement of the national regulations, and financial interests of community pharmacists as 63.8% of participants did not want to lose their customers. This financial attitude was noticed aprevious studies among Bulgarian and Lebanese community pharmacists (22, 23). On the other hand, feeling competent is the important factor affecting dispensing antibiotic without prescription by community pharmacists (24). In our study one of four participated pharmacists considers himself qualified to give the right medicine. The OTC selling antibiotic in community pharmacy may contribute to the increased resistance level recorded in Aleppo (25, 26). In the previous study conducting among Aleppo city population, 54% of adults have purchased antibiotic without prescription. Although the physician visit fees does not exceed 1000 Syrian Pound, some of them (26.4%) justified their behavior that they want to save their money as the pharmacist providing free medical help. However, 30% explained their action by shortage of time (27). 

The results of the study showed that the knowledge of participants about antibiotics resistance ranged from poor to good. Overall, the theoretical knowledge of participants about antibiotic resistance is inadequate. Despite 58% of pharmacist apparently have a good score, it should be noted that a half of the participants agreed to use antibiotic for common cold infections. This suggests that the pharmacistʹs knowledge may be affected by the fact that the majority ofclinicians were prescribed antibiotics too often in such conditions (28). 

It is a well-known fact that resistance to antibiotics has been linked to the consumption of drugs with evidence of a cause-effect relationship (29-32). However; confusion knowledge regarding antibiotics resistance phenomenon is obvious as only half of pharmacists gave the correct answer about statement «Even appropriate use of antimicrobials can cause resistance». The inadequate knowledge by pharmacistsʹ affect their attitude as (14.5%) of participants choose to buy a customer board spectrum antibiotic for tonsillitis treatment which is well known to affect the emergence of resistance to antibiotics (33). This inappropriate practice should be addressed in future campaign (34).

No difference was found between pharmacistsʹ total scores of knowledge other variable indicating that pharmacists in this study are concentrating their efforts to the work in a pharmacy, not to keep up with new information.

Among pharmacies chosen for study, there were 27 (13.8%) pharmacy working without a pharmacist and this finding is caused by concern. The staff of these pharmacies contributed to drug misused by providing misinformation about drugs and selling antibiotics according to popular demand (35, 36). In Saudi Arabia 28.6% pharmacists were unavailable at the designated hours in their pharmacies (37). In Pakistan, the situation was even worse and only about one-fifth of pharmacies sampled had unqualified personnel selling drugs and providing advice and treatment of common health problems (38).

One third of the pharmacists exhibited a good attitude toward counseling and directing patients although the information provide by pharmacist about is as important as the appropriateness of the medicines themselves. This was in agreement with Indian study which reported that 61% of participating pharmacists considered that patient counseling is their professional responsibility, but only few pharmacists were offering this service (39). However, a highly significant association has been found between pharmacists’ age, years of experience, socioeconomic location of the pharmacy, number of clinics near the pharmacy and between pharmacistsʹ attitude. The current study revealed that Syrian pharmacists like others in developing countries are still underutilized and their role as health care professionals is not enabled. These results corresponded with previous studies reported the important role of pharmacist in low income countries where people relied on the pharmacist advice in their health problem due to economic reasons (15, 40).

The surprising finding was that the majority of participating pharmacists declared disagreement about activation of law that restricts sales of antibiotics without prescription. This might be explained by the fact that activation the law would negatively affect the sales quantities at their pharmacies and reflects pharmacistsʹ commerce orientation even with the evidence of harm based practice. No association was found between sale of antibiotics without prescription conducted by participated pharmacists and their demographic characters, which reflect general behavior due to lack of strict control by the authorities.

The current study has several limitations. First, this survey included community pharmacists’ from only one city which limits its generalizability, further research is required to get the views of pharmacists in Syria. Second, as with all self-administered questionnaire based studies, there is the possibility that participants may over-report desirable behaviors or under-report undesirable behaviors. In addition, it may be possible that some respondents reading recommended materials and thus acquired a greater knowledge prior to questioning. As a result, the level of knowledge found in this study may be likely to represent a more favorable picture than would be observed. Howerver, despite the limitations described above, this study considered the first step for evaluating the pharmacistsʹ knowledge and attitude about antibiotic resistance which provides basic information for further studies. 

## Conclusions

This study reveals high rate prevalence of antibiotics dispensing without prescription which calls for imposing strict measures leading to restriction of antibiotic sales. 

The knowledge and attitudes of the pharmacists participate in this study indicate the urgent need for continuous awareness campaigns directed to pharmacists and public.

Educating the pharmacy staff on the risks of antibiotic resistant and emphasis on their role in helping to reduce the prevalence of resistance is an essential issue.

Pharmacists have to accept these challenges to ensure that antibiotics are used appropriately even during the self-medication. 

In addition, there is a need for improving pharmacy curricula in colleges, thereby building correct practice into the profession from the beginning and shifting from the concept of the pharmacy as a ‘shop’ and building a culture of pharmaceutical services.

## Funding

The study was conducted with in-house resources.
